# “I don’t want to take buprenorphine for the rest of my life”: Acceptance and Commitment Therapy for a Client Struggling to Reduce Low-Dose Buprenorphine (a Hermeneutic Single-Case Efficacy Design)

**DOI:** 10.1007/s11469-021-00729-2

**Published:** 2021-12-20

**Authors:** Kate Shepherd, Beth Pritty, Anna Tickle, Nima Moghaddam

**Affiliations:** 1 Trent Doctorate in Clinical Psychology, School of Psychology, University of Lincoln, Lincoln, UK; 2 Trent Doctorate in Clinical Psychology, Division of Psychiatry and Applied Psychology, University of Nottingham, Nottingham, UK; 3 Nottinghamshire Healthcare NHS Trust, Nottingham, UK; 4 Framework; Opportunity Nottingham; and Trent Doctorate in Clinical Psychology, University of Nottingham, B Floor, Yang Fujia, Jubilee Campus, Wollaton Road, Nottingham, NG8 1BB UK

**Keywords:** Acceptance and Commitment Therapy, Buprenorphine, Hermeneutic single-case efficacy design

## Abstract

The misuse of substances is often maintained by both physical and psychological factors. Opioid-substitution medications manage physical aspects of addiction; however, difficulties with emotional regulation and avoidance perpetuate continued substance misuse. In the UK, individuals who misuse substances are often excluded from mental health services, meaning these underlying difficulties are not addressed. Acceptance and Commitment Therapy (ACT) seeks to reduce emotional avoidance. A hermeneutic single-case efficacy design was used to evaluate the effects of ACT within drugs and alcohol service. Quantitative and qualitative data was critically analysed to understand factors involved in identified changes. Analysis recognised the client progressed towards two of three of their goals, related to motivation and anxiety. Their psychological flexibility also increased. ACT processes played a key role in this; however, the therapeutic relationship and psychopharmacological factors were also noted. Study limitations and clinical and research implications are discussed.

Addiction and substance dependency is a complex and significant issue facing the UK, with over a quarter of a million adults in contact with drugs and alcohol services (Public Health England, 2019). Drug addiction is defined as persistent and compulsive drug-taking (despite negative consequences) perpetuated by both biological and psychological components (Hasin et al., 2006). Substance use causes biological changes to the brain’s reward system, requiring repeated and/or increased doses to maintain allostasis and prevent unpleasant withdrawal symptoms (Cami & Farre, 2003). Understanding drug addiction via biological mechanisms alone, however, is reductionist, considering many individuals continue to take substances in the absence of withdrawal symptoms or craving (Everitt & Robbins, 2005).

While hedonistic effects or relief of withdrawal symptoms may reinforce drug-taking, increasing evidence suggests many individuals use substances primarily to regulate or avoid painful emotions (Weiss et al., 2014). Poor emotional regulation and avoidance are closely intertwined with mental health difficulties, and up to 86% of individuals who use substances also meet criteria for common mental health disorders (Weaver et al., 2003). Individuals who use substances and have common mental health problems are often excluded from primary care services due to substance dependency (Public Health England [PHE], 2017), despite contrary NICE guidance (NICE, 2007a). There is therefore a clear need for services to provide support to individuals who wish to cease maintenance therapy but struggle with underlying issues of emotional regulation and avoidance.

Poor emotional regulation is a risk factor for both initial and continued substance use (Cheetham et al., 2010; Shoal & Giancola, 2001). However, once emotional regulation and avoidance are addressed via psychological therapy, substance use and mental health conditions improve (Bahrami & Asghari, 2017; Conklin et al., 2015; Twohig et al., 2007). This suggests that without addressing emotional regulation, individuals will continue to use substances even if biological components are managed.

The National Institute for Health and Care Excellence (NICE) recommends pharmacological treatments, supplemented by psychosocial interventions for substance dependency (NICE, 2007a, b). Opioid dependence is treated using maintenance therapy, leading to abstinence supported by a prescribed substitute (such as buprenorphine, a semi-synthetic opioid; Johnson et al., 2005; Joint Formulary Committee, 2020). Maintenance therapy enables clients to manage withdrawal effects and reduces craving by providing a substitute and is empirically supported (Gossop et al., 2001). Consequently, maintenance therapies are considered “successful” due to their association with reduced illicit drug use (Sees et al., 2000).

Maintenance therapies, however, are not a viable long-term solution to substance dependency. Maintenance therapies do not address underlying issues with emotional regulation, meaning, once medications are ceased, problematic substance use may return and both clients and clinicians are reluctant to cease taking/prescribing medication (Cordella et al., 2017). This is problematic due to both the ongoing financial cost of prescribing and the continuous reinforcement that individuals need substances to cope (Joint Formulary Committee, 2020; Shamsalinia et al., 2017). Indeed, evidence has suggested that maintenance therapy may actually be detrimental in preparing individuals to cope with difficulties during abstinence (Compton et al., 2001; Zahari et al., 2016). It is clear, therefore, that providing concurrent maintenance therapy and psychosocial interventions, to address underlying psychological difficulties, would be most effective in treating substance misuse.

## Acceptance and Commitment Therapy (“ACT”)

ACT, a psychological therapy stemming from Cognitive Behavioural Therapy (CBT), proposes that psychologically painful experiences (such as grief and anxiety) are healthy and inevitable, and avoidance attempts result in inflexible, rigid behaviours and distress (Harris, 2019). ACT encourages psychologically “flexible” behaviour via three interconnected dyads: openness to experiences (OE), behavioural awareness (BA), and valued action (VA).

Each dyad is comprised of two core processes (which are polarised into psychologically inflexible and flexible terms). Definitions for each can be found in Table 1. Inflexible processes make it difficult for individuals to change and respond appropriately to situations—worsening overall wellbeing. Processes are only considered pathological, however, if they are maladaptive or problematic for the individual (Hayes et al., 2012). ACT focuses on skill development and understanding core processes, so that individuals can use techniques when needed to improve their wellbeing (Harris, 2019).

**Table 1:** Definitions of the core ACT processes (Harris, 2019; Hayes et al., 2012)

ACT dyad	Psychologically inflexible process (flexible term)	Definition
Openness to experiences	Experiential avoidance (acceptance)	Attempting to avoid psychologically painful experiences
	Cognitive fusion (defusion)	Taking cognitions as literal and unequivocal “truths”
Behavioural awareness	Lack of (present-moment awareness)	Preoccupations with the past or future
	Over-attachment to the ‘self-content’	A rigid and fixed view of “who” they are
Valued action	Remoteness from values (values clarity)	Limited understanding of what concepts are personally important
	Unworkable action (valued-committed action)	Action incongruent with one’s values

## ACT and Substance Dependency Treatments

Aspects of ACT, such as reducing avoidance and increasing present moment awareness, have been found to effectively reduce substance use via improving emotional regulation (Conklin et al., 2015; Tang et al., 2016). Several controlled trials report substance use reductions following ACT, alongside more sustained benefits than other therapy modalities for both wellbeing and substance use (Bahrami & Asghari, 2017; Lanza et al., 2014; Smout et al., 2010; Thekiso et al., 2015). ACT has been found to increase the effectiveness of opioid-substitute medications for polysubstance misuse compared to medications or other psychological therapies used alone (Hayes et al., 2004). This highlights ACT as a viable therapy alongside maintenance therapy for addressing the underlying emotional regulation and avoidance difficulties that face many individuals who use substances. It is important to recognise, however, supportive literature primarily originates from highly controlled trials, and while these could be argued to be reliable, lack of ecological validity may not reflect clinical practice. As discussed, the reality for many individuals is exclusion from psychological services—making it unclear how ACT would impact clients in a more realistic setting (PHE report, 2017).

## The Current Study, Service, Aims, and Hypotheses

The presented service is a publicly funded, multi-disciplinary, drugs and alcohol recovery support and treatment service, with no permanent psychological provision. Clients and clinicians have limited access to psychological professionals and instead primarily rely on pharmacological treatments as is typical for services in the UK, despite contradicting NICE guidance (PHE, 2017; NICE, 2007a). The service was provided with temporary access to a clinical psychology provision and used this to provide ACT to support to individuals who were struggling to cease their low-dose buprenorphine prescription due to underlying psychological difficulties.

This study presents a hermeneutic single-case efficacy design (HSCED): a mixed methods case study exploring possible links between therapeutic processes and outcomes while also evaluating possible non-therapy explanations for change (Elliot, 2015). This study aimed to explore the effectiveness of ACT for individuals who met the criteria for this additional provision, with specific focus on client-identified goals. To address this, the study explored the following: if personally meaningful change occurred for the client; if changes could be attributed to ACT; and what factors contributed towards changes.

## Method

### Design

A HSCED was used to meet the aims of the study: quantitative and qualitative data obtained from therapy in clinical practice was analysed to determine the factors contributing to any changes identified (Elliot, 2015).

The HSCED triangulated information to create an affirmative case (supporting the notion that therapy caused change) and a sceptical case (arguing no change or change did not occur due to therapy). Competing cases were then evaluated to assess and attribute client outcomes.

### Participants

#### Client

ACT was offered to eight service-identified individuals taking low, potentially sub-therapeutic, doses of buprenorphine who faced psychological barriers to ceasing buprenorphine. Two completed ACT; three declined on invitation (reasons unknown); and three disengaged after one, two, and three sessions, respectively (one due to bereavement). Therapy and data collection was completed as routine clinical practice therefore ethical approval was not sought. Both clients who completed ACT consented for their cases to be used for clinical research. Due to unforeseen circumstances (i.e. COVID-19), data from only one participant (pseudonym: “Bruce”) was accessible and is presented here. All identifying details have been altered to protect anonymity.

#### Therapist

ACT was delivered by a final year Trainee Clinical Psychologist who received weekly supervision from a qualified Clinical Psychologist experienced in ACT (the “supervisor”). The therapist kept weekly clinical notes but was not involved in data analysis.

#### Researcher

The lead author was a second year Trainee Clinical Psychologist with experience using ACT therapeutically and minimal experience working with clients experiencing substance dependency and was independent to both the therapy and service. HSCED analysis typically includes an additional independent critique. Independent individuals were unavailable; therefore, steps were taken to reduce bias (see “Data analysis”).

### Measures

Pre-therapy data was collected, but no extended baseline was established for any measure due to the ethical implications of withholding treatment. Instead, longevity of problems was determined during the assessment process and was reportedly longstanding.

Measures were chosen in line with the principles of ACT. As ACT is a transdiagnostic approach that only considers processes pathological if they are problematic for the individual, the Personal Questionnaire (PQ) is a Client-Generated Outcome Measure (Elliott et al., 2016) that allows the participant to specify problems they wish to address through therapy and rate how problematic each has been during the past week. This idiographic psychometric arguably allows a more meaningful measure of the “effectiveness” of therapy from the client perspective. A reduced score on the PQ indicates client improvement. The Session Rating Scales is a brief measure designed to assess the client rating of therapeutic alliance, which is one of the best predictors of therapy outcome (Duncan et al., 2003). Tracking alliance session by session allows the therapist to attend to alliance and respond to any issues raised in a timely manner. The Comprehensive Assessment of Acceptance and Commitment Therapy (CompACT 8) was used as it measures the six core processes that constitute psychological flexibility, where an increased score indicates improvement. Albeit this concept and its measurement are not without problems, psychological flexibility is the proposed mechanism of action in ACT and associated with greater wellbeing and the ability to pursue valued goals, even in the face of challenges (Doorley et al., 2020). Finally, the Short Warwick-Edinburgh Mental Wellbeing Scale (SWEMWBS) was used as a brief overall measure of wellbeing, with a focus on strengths, assets, and recovery, which is more in keeping with ACT principles than a measure of any specific symptoms. An increase in score on the SWEMWBS indicates improvement (Table 2).

**Table 2:** Psychometric measures used

Measure	Construct	Item details and directionality	Psychometric properties
Short Warwick-Edinburgh Mental Wellbeing Scale (SWEMWBS; Warwick Medical School, n.d.a)	Wellbeing	7 items; 5-point Likert scale; higher scores indicate more positive wellbeing	Good internal reliability (*α* = 0.84) and high correlation with related measures and the original 14-item version (Ng Fat et al., 2017; Stewart-Brown et al., 2009); Test–retest reliability data is unavailable, however the original WEMWBS reliability is high (*r* = 0.83; Tennant et al., 2007)
Session Rating Scale (SRS; Duncan et al., 2003)	Therapeutic relationship between therapist and client	4 items; clients mark their agreement with each item on a line to create a centimetre measurement; higher scores indicate a more positive therapeutic relationship	Correlates highly with similar measures, has good internal reliability (*α* = 0.88) and encouraging test-rest reliability (*α* = 0.64; Duncan et al., 2003)
Comprehensive Assessment of Acceptance and Commitment Therapy (CompACT-8; Morris, 2019)	Psychological flexibility (total score), with subscales clustering OE, VA, and BA processes	8 items; 7-point Likert scale; higher scores indicate higher psychological flexibility both within each subscale and as a total	Good internal validity (*α* > 0.70) and correlates significantly with similar measures of ACT processes (Morris, 2019)
Personal Questionnaire (PQ; Elliott et al., 2016)	Client-identified problems stated as goals for therapy	Up to 10 client-generated statements; 7-point Likert scale; higher scores indicate greater problems	Good internal reliability *α* = 0.80 to 0.77 and temporal reliability *α* = 0.57 (Elliott et al., 2016)

### Procedure

A pre-therapy meeting with the therapist introduced Bruce to ACT and established his expectations for therapy. Bruce attended 12, weekly, 45-min-long sessions of ACT. Session one consisted of measure completion, history taking, and goal setting (including PQ problem statements).

ACT is non-linear and process-driven; specific session content is reactive to the difficulties clients bring to each session. Bruce’s sessions closely followed an ACT protocol developed by the therapist and supervisor and taught techniques surrounding each ACT process.

Bruce completed the PQ, SRS, and CompACT-8 during each session and completed the SWEMWBS) during the first and last session.

Buprenorphine dosage was monitored throughout.

#### Change Interview

A post-therapy, semi-structured change interview (CI) was conducted by the supervisor independently to promote candid feedback (Elliot, 2006). Questions are outlined in Table 3.

**Table 3:** Change interview schedule

1. General questions (5 min) 1a. How are you doing now in general? 1b. What has therapy been like for you? How has it felt to be in therapy? 1c. What medications on your currently on? (dose, how long, last adjustment, herbal remedies)				
2. Changes (10 min) 2a. What changes, if any, have you noticed in yourself since therapy started? (Interviewer: reflect back change to client and write down brief versions of the changes one per change sheet. Optional follow-up questions: “Are you doing, feeling or thinking differently from the way did before?” “What specific ideas, if any, have you got from therapy, including ideas about yourself or other people?” “Have any changes been brought to your attention by other people?” 2b. Has anything changed for the worse since therapy started? 2c. Is there anything that you wanted to change that hasn’t since therapy started?				
3. Change ratings (10 min) [see separate change sheet with rating scales a, b and c) 3a. For each change, please rate how much you expected it vs. were surprised by it? 3b. For each change, please rate how likely you think it would have been if you hadn’t been in therapy? 3c. How important or significant to you personally do you consider this change to be?				
4. Helpful aspects (10 min) Can you sum up what has been helpful about therapy? Please give examples (e.g. general aspects, specific events)				
5. Attributions (5 min): In general, what do you think has caused the various changes you described? What do you think might have brought them about, including things both outside of therapy and in therapy?				
6. Resources (5 min): 6a. What personal strengths do you think have helped you make use of therapy to deal with your problems? (what you’re good at; personal qualities)? 6b What things in your current life situation have helped you make use of therapy to deal with your problems? (family, relationships, living arrangements)				
7. Problematic aspects (5 min) 7a. What kinds of things about the therapy have been hindering, unhelpful, negative, or disappointing for you? (general aspects of specific events) 7b. Were there things in therapy which were difficult or painful but still OK or perhaps helpful? What were they? 7c. Has anything been missing from treatment? (What would have made therapy more effective or helpful?				
8 Limitations (5 min) 8a. Are there things about you that you think have made it harder for you to use therapy to do deal with your problems? If so, what? 8b. Are there things in your life situation that have made it harder for you to use therapy to deal with your problems? (family, relationships, living arrangements etc.)				
9. Suggestions (5 min) Do you have any suggestions for us, regarding the research or the therapy?				
Do you have anything else that you want to tell me?				
10. Reflecting on changes shown on session measures: We have put together the scores from all of the questionnaires you filled in during therapy. I would like to talk through them and as I do get your view on the results, for example whether you think they are in line with your expectations or not * Personal questionnaire:* this is the measure that you used to label problems you wanted to work on and rated how much they bothered you. A lower score suggests the problem bothers you less. The dark line shows before you started therapy; the lighter line shows your scores on your last session. Where there is an * this is considered clinically significant change, e.g. good * CompACT:* This is the 8-question measure of what we call psychological flexibility. A higher score indicates better psychological health. These are your scores from the start to the end of therapy The CompACT breaks down into three sections (other side of sheet): behavioural awareness, openness to experience, and valued action, i.e. what you do that is in line with how you want to live. These graphs show your scores from the start to the end of therapy on the three scales. Higher scores indicate better psychological health * The SWEMWBS:* This is the Short Warwick-Edinburgh Mental Wellbeing Scale that you completed just before and after therapy. The * indicates clinically significant change. A higher score shows greater wellbeing * The Session Rating Scale:* This is the measure you completed at the end of each session, about your experience of the session. A higher score indicates better working alliance with the therapist				
Change/s ratings sheet: (Complete one sheet per identified change)				
What was the change?				
a. Please rate how much you expected it vs. were surprised by it:				
1 Very much expected it	2 Somewhat expected it	3 Neither expected nor surprised by it	4 Somewhat surprised by it	5 Very much surprised by it
b. Please rate how likely you think it would have been if you had not had therapy?				
1 Very unlikely without therapy (clearly would not have happened)	2 Somewhat unlikely without therapy (probably would not have happened)	3 Neither likely nor unlikely (no way of telling)	4 Somewhat likely without therapy (probably would have happened)	5 Very likely without therapy (would have happened anyway)
c. How important or significant to you personally do you consider this change to be?				
1 Not at all important	2 Slightly important	3 Moderately important	4 Very important	5 Extremely important

#### Adherence

ACT-adherence was monitored throughout by the supervisor via clinical supervision as part of routine clinical practice and was reported to be adequate.

### Analysis

Anonymised data collected for analysis included a case vignette (including history and referral information), clinical notes, and psychometric data.

#### Case Record

Bruce’s case was summarised into a narrative report by the therapist, providing the researcher context for analysis, including history, formulation, and therapeutic goals.

#### Quantitative Analysis

Reliable change (RC) computations were applied to examine the statistical reliability of quantitative changes (Jacobson & Truax, 1991). Changes meeting criterion for RC were further assessed in terms of clinical importance—i.e. whether the RC represented a Clinically Significant Change (CSC; a reliable change that crosses the caseness threshold, moving between clinical and non-clinical ranges of scoring). Thus, CSC in the direction of *improvement* was demonstrable where (1) pre-therapy score was in the clinical range, (2) change from pre- to post-therapy met criterion for RC, and (3) post-therapy score was in the non-clinical range. RC and CSC criteria were established based on data from Ng Fat et al. (2017), Morris (2019), and Elliott et al. (2016) for the SWEMWBS, CompACT-8, and PQ, respectively. Caseness was determined by SWEMWBS scores < 21 (Warwick Medical School, n.d.b) and PQ scores > 3 (Elliott et al., 2016). Caseness data were unavailable for the CompACT-8; therefore, Jacobson and Truax’s “criterion a” was used to tentatively estimate thresholds (Jacobson & Truax, 1991).

Outcome data collected weekly (PQ, CompACT-8, including subscales) was plotted and examined via visual analysis to explore anomalous data and trends.

#### Qualitative Data

Clinical process notes and CI information were collated to explore changes that may not be captured by quantitative psychometrics, such as those not measured by the psychometrics or personally meaningful changes. Clinical process notes were prepared for use within the HSCED analysis by removing data that appeared unrelated to change or change processes; the researcher read clinical notes and highlighted salient points that appeared related to change. Highlighting was conducted both prior and post quantitative analysis to facilitate inductive and deductive considerations of change. This process was conducted by the researcher and independent of the therapist to reduce bias. The abridged clinical notes and CI answers were then used to provide narrative evidence within the later HSCED analysis (see below).

#### HSCED Analysis

To be considered indicative of “change”, evidence was required to meet quantitative criterion for CSC (where appropriate) and be corroborated by qualitative data (CI or therapist notes). Similarly, at least two sources of evidence were required to support therapy-change links (retrospective client attributions, via the CI, and visual analysis correlating changes on process and outcome measures; Elliot, 2015).

Complementary and contradictory evidence were then triangulated using guidance by Elliot (2015) to create two cases:

1. Affirmative, including if change occurred, if the client attributed changes to therapy, if changes were personally meaningful, and if changes and the processes of therapy appeared to be linked
2. Sceptical: exploring the idea of no change, negative change, or other feasible reasons for positive change (e.g. therapeutic relationship/relational factors, extra-therapy factors, or psychobiological causes)

The affirmative case had a rebuttal opportunity, and then both cases were evaluated via adjunction. This is typically, but not necessarily, completed by an independent party. Due to unavailability of independent individuals, however, steps were taken to otherwise minimise bias. The lead author was independent to the therapy and service. Analysis was reviewed by the supervisor and a clinical psychologist independent of the therapy and service. To improve reflexivity, a reflective journal was kept throughout analysis while developing the affirmative and sceptical interpretations, enabling the lead author to consider both cases simultaneously. This also mitigated possible bias towards promoting the ACT model due to the researcher’s prior experiences. Finally, the triangulation process was iterative to accommodate reflective journal notes. Further detail regarding HSCED analysis may be found in guidance by Elliot (2015).

## Results

### Case Record: “Bruce”

Bruce (40 years) entered the service 2 years ago. Bruce began misusing opiate-based prescription medication 3 years ago following the breakdown of a long-term romantic and subsequent loss of his home and regular contact with his children. More recently, Bruce also began using alcohol and self-harm (cutting) to cope. Bruce had historically attempted buprenorphine abstinence, however restarted medication citing considerable psychological distress and “overwhelming feelings of sadness”. Bruce reported engaging in low-intensity CBT, and despite Bruce saying this was “somewhat helpful”, Bruce still felt he was dependant on substances to cope.

At the start of therapy, Bruce lived in a temporary accommodation hostel and had a difficult relationship with his ex-partner. He had limited access to his children and experienced social anxiety following the comments and actions of his ex-partner, meaning he avoided socialising with friends and family.

It was formulated that Bruce used opioids and alcohol to avoid painful psychological experiences, resulting in withdrawal from meaningful activities. Practical concerns prohibited Bruce from engaging with his children—his lack of adequate housing meaning they could not visit him.

Bruce’s self-identified goals (via the PQ) were wanting to improve his:

- Goal 1. Poor motivation “to do anything”
- Goal 2. Housing situation (his children could not stay the night)
- Goal 3. Anxiety (particularly social anxiety)

### Determining if Change Occurred

#### Psychometric Quantitative Data

Bruce rated the SRS as maximal every session; therefore, no statistical analysis was conducted.

All scales except PQ goal 2 exhibited RC. Pre-therapy scores for some measures (PQ goal 2, SWEMWBS, and CompACT BA and OE subscales) did not meet caseness; therefore, CSC (in the direction of improvement) was not demonstrable for these measures (Table 4). Measures demonstrating CSC were PQ goals 1 and 3, overall psychological flexibility (total sum of CompACT-8 subscales), and VA. It is important to recognise that these quantitative figures are merely indicative of CSC and qualitative data is also important to confirm personally meaningful changes.

**Table 4:** Bruce’s pre- and post-therapy psychometric scores

Measure (subscale)	Clinical cut off^1^	RC required^2,3^	Pre-therapy	Post-therapy	Change (**bold** indicates CSC)
SWEMWBS	< 21	1(↑)	23	24	+ 1*
**PQ goal 1 (Poor motivation)**	** > 3**	**2(↓)**	**6**	**2**	-4*
PQ goal 2 (Housing)	> 3	2(↓)	3	3	+ 0
**PQ goal 3 (High social anxiety)**	** > 3**	**2(↓)**	**6**	**2**	-4*
**CompACT-8 total**	** < 16**^**4**^	**5(↑)**	**14**	**33**	+ 19*
CompACT-8 (BA)	< 1^4^	2(↑)	4	9	+ 5*
CompACT-8 (OE)	< 2^4^	2(↑)	3	9	+ 6*
**CompACT-8 (VA)**	** < 9**^**4**^	**3(↑)**	**7**	**15**	+ 8*

^1^Threshold for determining caseness

^2^Minimum change to indicate statistically reliable change

^3^Arrows (↑/↓) indicating direction of change for apparent client improvement

^4^Estimated caseness. *Meets criterion for statistically reliable change (RC) at *p* < .05

**Fig. 1:**
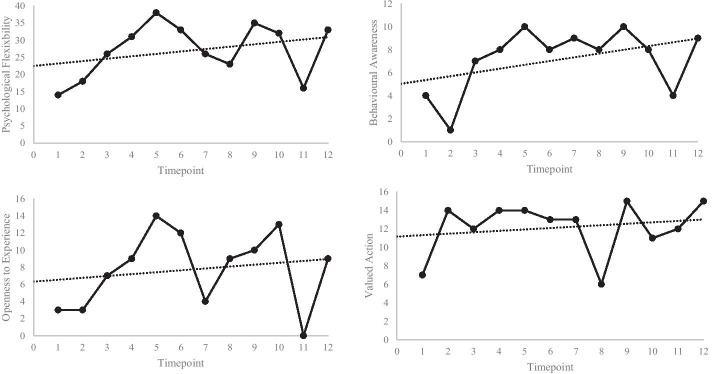
Weekly ratings for the sum of CompACT-8 subscales (top left); BA (top right); OE (bottom left); and VA (bottom right). Higher scores indicate increased psychological flexibility

**Fig. 2:**
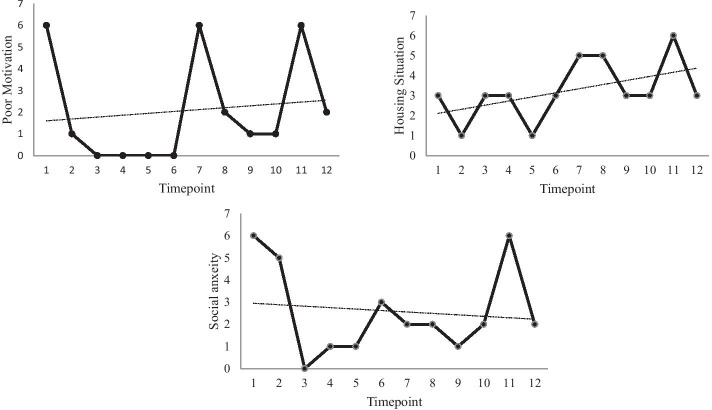
Weekly ratings for the three PQ goals. Higher scores indicate greater difficulties

Overall psychological flexibility and each CompACT-8 subscale showed an upwards trend throughout therapy. Week 11 for all scales, week 7 for psychological flexibility and OE, and week 8 for psychological flexibility and valued action, however, showed lower scores (Fig. 1). Regarding PQ measures, only goal 3 (social anxiety) showed a weekly trend indicative of improvement (Fig. 2).

#### Buprenorphine Dosage

Buprenorphine dosage increased: beginning at 0.4 mg daily and increasing between sessions three and four to (and remaining at) 0.8 mg daily indicating dose changes from sub-therapeutic to a very low dose (which may still be sub-therapeutic; Joint Formulary Committee, 2020). Unfortunately, due to service changes resulting from the impact of the COVID-19 pandemic, it was not possible to gather monitoring or follow-up data regarding Bruce’s buprenorphine dosage.

#### Salient Notes

Examples of change within the therapist’s clinical notes primarily referenced improved motivation to engage in meaningful activities. From session three onwards, Bruce increasingly discussed “keeping [himself] busy” with more varied activities as therapy progressed (including socialising, seeing his children, and sports). During the final session, Bruce reported seeking lifelong learning opportunities and to return to work. Bruce cited increased motivation and activity as reasons for “feeling better within [himself]” (session five); his reduced alcohol consumption (session five); and had encouraged him to seek and commence anti-depressant medication from his GP (session six).

Salient notes highlighted that Bruce changed the way he perceived and responded to difficult situations. Bruce reported realising he used drugs and alcohol to mask unprocessed feelings, which was why he found reducing his buprenorphine difficult (session two). Bruce referenced several challenging situations during therapy and noted being surprised by his different responses (“I didn’t get as upset as I normally would”) and did not self-harm despite urges to do so (session eight). Bruce reported he ruminated less and had commenced and continued meaningful activities despite barriers (e.g. outdoor activities with children which do not require appropriate housing). During the final session, Bruce reported attempting to live a meaningful life despite continuing practical difficulties (like inadequate housing).

#### Change Interview

Bruce reported finding therapy useful to “have someone to talk to” and to learn distress-management strategies. Particularly, Bruce referenced finding it useful to set concrete goals between sessions (“[the therapist] sent me away with something to achieve”).

Bruce noted six changes following therapy (Table 5). Bruce said he had learnt from ACT to not “beat yourself up and lie in bed waiting – it won’t change anything”. This was Bruce’s interpretation of “creative hopelessness”, an ACT activity which encourages recognition of current unhelpful behaviour and promotes change (Harris, 2019).

**Table 5:** Changes Bruce reported during the CI

Bruce-identified change	How expected/surprising change was	How likely the change would have occurred without therapy	Importance of change
Learnt ways to deal with (difficult or painful) things using techniques learnt in therapy	Very much expected	Very unlikely	Moderately important
Learnt to socialise more	Very much expected	Very unlikely	Extremely important
Seeing children much more often	Very much expected	Very unlikely	Extremely important
Drinking less	Neither expected nor surprised	Neither likely nor unlikely (no way of telling)*	Extremely important
Not self-harming (used to)	Very much surprised	Neither likely nor unlikely (no way of telling)*	Extremely important
Not “stressed out” and crying as much	Somewhat expected	Somewhat unlikely	Very important

^*^Bruce suggested commencing anti-depressant medication may have impacted this but was unsure

### Determining What Produced Change

#### Affirmative Case

The affirmative case identified three changes. Bruce expressed these were longstanding difficulties and reported no other significant extra-therapeutic factors that contributed towards these changes, preliminarily suggesting change was caused by ACT.

1. Increased motivation and valued committed action
  Clinical notes and the CI revealed Bruce reported increased motivation and engaged in more personally meaningful activities. Quantitative evidence demonstrated CSC for PQ (for which a reduced score indicates improvement) goals regarding “motivation” and “social anxiety” and for VA (Table 4) which, within the context of qualitative reports, suggests these were clinically significant and personally meaningful changes. CI indicated Bruce believed these changes were unlikely to have occurred without therapy and gradual quantitative increases in VA (for which an increased score indicates improvement) (Fig. 1) suggest ACT processes were linked to these changes.
2. Improved ability to cope with difficult experiences
  Quantitative data and self-report showed Bruce experienced distress throughout therapy, however still increased his engagement in meaningful activities—something Bruce reported being unable to do prior to therapy. Bruce also identified “learning to deal with difficult things” as a change unlikely to have occurred without therapy during the CI.
  One example of this is Bruce’s use of self-harm. During early sessions, Bruce reported self-harming between sessions to cope with psychological distress. During session 11, however, Bruce reported several ACT techniques he used to sit with self-harm urges and prevent this. Reported techniques centred around VA and that week, while BA and OE scores decreased (indicating decline rather than improvement), and VA remained high (Fig. 1). It would therefore appear that ACT processes caused a change in how Bruce coped with difficult experiences.
3. Increased psychological flexibility

Quantitative evidence showed psychological flexibility gradually, yet reliably and significantly, increased throughout therapy, as demonstrated by increased CompACT scores (Table 4; Fig. 1). Bruce also reported several examples of becoming more psychologically flexible within qualitative data; Bruce described incidents where he would previously have become frustrated but instead reacted differently. Bruce did not comment on increased psychological flexibility during the CI, but did report that the most helpful aspect of therapy was an ACT-specific technique. This, alongside other changes discussed and the gradual nature of these changes across therapy, suggests ACT was instrumental in these changes.

#### Sceptical Account of Change

Some changes are apparent, yet SWEMWBS data indicated no changes to wellbeing—typically a core therapy aim. Due to lack of follow-up data, there is also no evidence to suggest the described changes are lasting—making it difficult to confidently argue these changes are meaningful. Finally, Bruce’s buprenorphine dosage increased during therapy (although reasons for this are unclear). Therefore, it may be argued that Bruce’s changes during therapy were either unimportant (as they did not improve wellbeing nor is there evidence of longevity) or potentially negative (increasing buprenorphine).

Changes may also be due to psychopharmacological effects. Bruce increased buprenorphine dosage and also commenced anti-depressant medication during therapy. This may have increased Bruce’s motivation to engage in activities and subsequently caused the other changes discussed, with the gradual increase being attributed to an initial placebo effect then medication taking effect (following the latency between medication commencement and medication therapeutic effect). This presents an alternative explanation of change, external to ACT processes.

Exploring relational factors, Bruce’s apparent improvements during session 11 may be due to “hello-goodbye” effects (Elliott, 2015)—artificially improving final psychometric scores to maintain the therapeutic relationship and justify one’s own engagement in therapy. Bruce rated the SRS maximally throughout, suggesting Bruce felt influenced (consciously or unconsciously) to provide a socially desirable response to please the therapist. Bruce also reported minimal instances of coping poorly, including when a homework task specifically requested examples of this (possibly reporting an overly positive view of therapy to please the therapist). This suggests changes may due to relational artefacts rather than meaningful, lasting change.

Finally, generic therapeutic techniques, rather than ACT processes specifically, may have caused change. During the CI, “having somebody to speak to” and “concrete goals” were noted to be useful—hinting that change was instigated by common and non-specific therapeutic techniques. Behavioural activation is a CBT technique aimed to improve activity levels and may have caused similar changes (Chartier & Provencher, 2013). Therefore, changes may have occurred, but not due to ACT processes.

#### Affirmative Rebuttal

While Bruce altered his medication during therapy, it is notable that the discussed changes appeared prior to this and that medication may have taken longer to take affect than is evidenced in Bruce’s data. Alternatively, therapy may have facilitated change which enabled Bruce to access these medications: Bruce had not commenced anti-depressant medication prior to ACT despite long-term difficulties. This could be evidence of Bruce responding more flexibly to his difficulties (seeking novel solutions)—further supporting the notion ACT processes instigated positive change.

Relational factors may have facilitated change, but the “hello-goodbye” effects discussed above may be misleading. Session 11 saw stark reductions across all psychometrics, and Bruce reported several difficult events. Although the following (final) week scores improve dramatically, if session 11 is considered anomalous and omitted, the effect disappears—suggesting the “hello-goodbye” effect is not present.

Bruce also referenced several factors unrelated to the therapist which contributed towards change. The change interview was completed by an independent party, and therefore, the risk of Bruce responding in a socially desirable way was mitigated, adding to the evidence supporting ACT playing a key role in instigating change.

Lastly, although changes may be due to general therapeutic techniques, Bruce also reported previously undergoing CBT. This CBT would likely have included general therapeutic techniques, and Bruce reported this was not effective in helping him manage difficult experiences. The CI, however, noted ACT had taught Bruce “ways to deal with difficult things”—suggesting ACT (rather than general therapeutic techniques) had caused the aforementioned changes.

### Adjudication

Personally meaningful change did appear to occur for Bruce, however not in all areas (i.e. wellbeing). ACT was likely a key factor in changes, but other contributing factors were also apparent. Particularly, the therapeutic relationship seemed to play a key role, and Bruce highlighted this in the CI. Bruce also underwent medication changes, meaning psychopharmacological factors cannot be ruled out. Despite this, results suggest changes began prior to medication changes and commencing medication may be an addition ACT-instigated change.

## Discussion

The study aimed to explore the effectiveness of ACT for individuals who were struggling to cease their low-dose buprenorphine prescription due to underlying psychological difficulties, focusing on the following: presence of meaningful change; if changes were attributed to ACT; and what factors contributed to changes. The study found that Bruce underwent three meaningful changes: increased motivation and meaningful activity engagement; increased ability to cope with difficulties; and increased psychological flexibility. Adjudication determined that changes were primarily due to ACT processes. Additional factors included the therapeutic relationship and psychopharmacological effects, yet changes began prior to psychoactive medication changes and Bruce outlined several, non-relational, ACT-specific factors which he believed caused change. The aims of the study were therefore met.

Both psychopharmacological and ACT processes contributed to the changes Bruce experienced. Wider literature suggests these factors may be interlinked, and previous evidence has found ACT is most effective when used alongside maintenance therapy and enhances the effects of this (Hayes et al., 2004). It may be that, while opioid-substitution medication is required to manage the acute difficulties of withdrawal, ACT addresses the underlying psychological difficulties perpetuating substance misuse (Cami & Farre, 2003; Smout et al., 2010). Bruce became more able to manage emotional distress—supporting this notion and adding to the literature demonstrating ACT is a viable method of addressing the perpetuating factors of substances use.

## Limitations and Strengths of the Study

Limitations of the study surround the outcome measures. Therapy focused on client-identified goals, yet Bruce’s goal of improving his housing situation may have been unachievable using ACT. ACT-congruent goals must be maladaptive, whereas housing-dislike may have been adaptive by motivating Bruce to seek better accommodation to enable engagement in further value-committed actions. Measuring the effectiveness of ACT according to this goal may therefore be inappropriate and present a misleading view of said effectiveness. Similarly, ACT aims to reduce distress and, consequentially, improve wellbeing (Harris, 2019). Bruce’s static wellbeing score may therefore appear to undermine the effectiveness of ACT, yet Bruce’s wellbeing score was above the non-clinical population mean and did not meet caseness (Warwick Medical School, n.d.b). Bruce’s wellbeing may have encountered a ceiling effect which accounted for lack of improvement, rather than being evidence against ACT’s effectiveness.

A further limitation related to outcomes could be the increase in buprenorphine dosage during treatment and lack of information regarding follow-up in this regard. Considering the context of the study is a substance use treatment service, it could be postulated that an overall measure of success would be if Bruce had been able to stop using buprenorphine completely. However, this did not occur during the ACT intervention and information as to whether it was achieved later is not available.

Study improvements could include closer ACT fidelity monitoring. Sessions followed a protocol and ACT-adherence was supervised, yet fidelity was reliant on therapist self-report. Some comments within clinical notes cast some doubt on fidelity; Bruce was reported to express something was good because he “does not have to sit with the anxiety”—incongruent with ACT. Despite this, many other examples demonstrate ACT-congruence. This suggests that while adherence was likely high, monitoring fidelity more closely (such as audio-recording sessions) would improve the study.

An important strength of the study is that data analysis was independent of the service and therapy. All analysis was conducted by an independent researcher unaffiliated with the service or therapist—reducing bias towards promoting a positive view of therapy. Possible bias towards ACT by the researcher was noted, and a reflective log was kept, to improve reflexivity and mitigate bias. Results were reviewed by the supervisor and a clinical psychologist independent of both the service and therapy. The steps taken to reduce bias add credence to the study outcome which supports the use of ACT to support individuals who misuse substances.

## Clinical Implications for the Service

NICE guidance recommends providing support to individuals who both misuse substances and have co-occurring mental health difficulties; however, many find themselves excluded from services and underling mental health difficulties are unsupported (NICE, 2007a; PHE, 2017). The discussed service has taken positive steps to support individuals who are typically excluded from services, and for Bruce, ACT has had a positive impact on his mental health and NICE guidance has been met. It was also reported the second individual who completed ACT expressed subsequent benefits; however, lack of analysis means the reasons surrounding this cannot be attributed to ACT. Within the discussed service, it is therefore suggested that ACT has a positive impact of individuals who complete it.

It must be noted, however, that six individuals were offered ACT but did not complete this—initially suggesting psychotherapy is not preferential within the service. This potentially has service implications for continuing to offer ACT. Importantly, it is expected that around 20% of individuals will terminate psychotherapy early regardless of modality (Swift & Greenberg, 2012). Furthermore, three individuals declined without further information. The reasons for declining and non-completion are unclear from the data provided, and therefore, inferences cannot be made regarding reasons for this. It would be useful to continue to offer ACT and monitor uptake/non-completion and reasons surrounding this over time. This will enable a more complete understanding of the need for ACT for this service.

## Conclusions and Recommendations for Future Research

Bruce expressed that he had learnt techniques to better tolerate difficult situations. This adds to the increasing literature demonstrating the usefulness of ACT in improving emotional regulation and reducing emotional avoidance. Previous literature has demonstrated ACT to be effective in improving psychological flexibility by improving the ACT process OE, which then subsequently reduces substance use (Bahrami & Asghari, 2017; Lanza et al., 2014). Additional literature has also discovered similar findings, however improved substance use by teaching BA techniques (Conklin et al., 2015; Tang et al., 2016). Bruce also demonstrated an improvement in psychological flexibility, however improved primarily via the VA process. This suggests that ACT aids individuals who misuse substances via improving their psychological flexibility; however, the ACT processes that are involved with this may be different. Future research may benefit from conducting a detailed case series including an ACT process analysis on multiple individuals, to explore the reasons why different individuals may improve in psychological flexibility via different ACT processes.

Further research may also seek to explore the longevity of changes that occur following ACT therapy. An argument proposed by the sceptical case highlighted that it is difficult to tell if the changes Bruce underwent were lasting due to the lack of follow-up (and therefore if ACT created meaningful changes). Therefore, the literature base may benefit from research exploring longitudinal data regarding the changes individuals experience following psychological therapy.

The study found, for Bruce, ACT resulted in increased motivation and engagement in meaningful activities, increased ability to cope with difficulties, and increased psychological flexibility—although relational and psychopharmacological factors also influenced these changes. Despite some limitations regarding outcome measurement, the study adds to the literature supporting the use of ACT for those who struggle with the psychological factors perpetuating substance misuse. Offering ACT ensures the discussed service meets NICE guidance; further research exploring reasons for uptake and non-completion would enable the service to better understand the preference for psychotherapy; however of those that completed ACT, it is clear that ACT was beneficial. Wider psychological research may also benefit from exploring how changes are influenced by individual differences in ACT processes and long-term impact of therapy.
